# Can the Behavioural Spillover Effect Affect the Environmental Regulations Strategy Choice of Local Governments?

**DOI:** 10.3390/ijerph18094975

**Published:** 2021-05-07

**Authors:** Yaling Deng, Daming You, Yang Zhang

**Affiliations:** 1 School of Business, Central South University, Changsha 410083, China; dengyl@csu.edu.cn (Y.D.); youdaming2016@163.com (D.Y.); 2 School of Management, Hunan University of Technology and Business, Changsha 410205, China

**Keywords:** environmental regulation, spillover effect, green technology innovation, evolutionary game

## Abstract

Combined with the characteristics of the Chinese environmental regulation supervision system and evolutionary game theory, the spillover effect of local governments’ investment behaviour has been incorporated into their payment function to study the influence of spillover on the strategy choice of local governments and enterprises. The results show that (1) the spillover effect is one of the reasons for distortions in the implementation of environmental regulations. Whether the influence of the spillover effect on the probability of local governments choosing the strategy of strict supervision is positive or negative depends on the environmental benefit of the local government’s environmental protection investment. (2) Increasing the reward for the enterprise’s complete green technology innovation behaviour is conducive to improving the probability of the enterprises choosing the strategy of complete green technology innovation, while it reduces the probability of local governments choosing the strategy of strict supervision. Increasing punishment for enterprises’ incomplete green technology innovation behaviour is conducive to improving the probability of enterprises choosing the strategy of complete green technology innovation, but its impact on the probability of local governments choosing the strategy of strict supervision is uncertain due to the limitations of many factors. (3) Enterprises’ emission reduction capacity is positively related to the probability of the enterprises choosing the strategy of complete green technology innovation and is negatively related to the probability of local governments choosing the strategy of strict supervision. The research conclusions provide a new explanation for the distorted enforcement of environmental regulations from the perspective of the spillover of local governments’ investment behaviour.

## 1. Introduction

Since reform and opening up, China’s economy has grown by leaps and bounds and has become the world’s second largest economy after the United States. However, with rapid economic expansion, China’s ecological environment has been seriously damaged. In the face of the dual pressure of environmental protection and economic development, the 19th National Congress of the Communist Party of China proposed adhering to the basic national policy of ‘saving resources and protecting the environment’ and emphasized that green technology innovation is a major measure to promote the transformation of the production mode of enterprises and to realize the win-win situation of economic benefits and environmental effects. Green technology innovation refers to a series of technologies in the production process, such as improving the production process, product structure, and innovative production activities, which are conducive to reducing energy consumption, reducing pollutants and improving production efficiency [1]. Since enterprises’ green technology innovation behaviour increases their production costs, it may not produce direct economic benefits and has the ‘dual externality’ of environmental benefit spillover and knowledge technology spillover [2]. Enterprises that conduct green technology innovation pay all the costs of innovation activities but cannot obtain all (or even most) of the benefits of innovation. Therefore, the motivation for enterprises to choose green technology innovation comes from the trade-off between costs and benefits as well as the uncertainty and effectiveness of R & D activities. This kind of unequal return on investment leads to the widespread phenomenon of ‘free riding’ in the market, and the market mechanism alone cannot effectively promote enterprises’ green technology innovation behaviour. Hence, government policy intervention is an important guarantee to promote the green transformation and upgrading of enterprises. The policy pressure imposed by regulators is, to some extent, conducive to stimulating enterprises’ environmental responsibility and helping them increase their research and development (R&D) efforts [3,4]. Although the central government has formulated a series of environmental policies, such as sewage charges and innovation subsidies, the environmental regulation efficiency is still low for that local governments maximize their own interests by reducing enforcement, providing tax incentives, lowering environmental standards, etc. [5].

China’s environmental policies are formulated by the central government and implemented by local governments, and there is information asymmetry in this principal–agent relationship. Local governments in various regions are motivated to distort the enforcement of environmental regulations, such as reducing enforcement, providing tax incentives and lowering environmental standards [6]. However, against the background of ‘economic decentralization and political centralization’, local governments are facing the dual pressure of economic growth and political promotion, so they compete with regard to the promotion evaluation index, public goods supply, and the introduction of liquid capital [7]. As the direct supervisor of enterprises’ production activities, local governments’ choice of competitive strategies plays an important role in guiding and motivating enterprises’ green technology innovation behavior [8].

The existing literature focuses on the influencing factors of the game mechanism of environmental regulations between local governments and enterprises, but few studies have considered the spillover effect of local governments’ investment behaviour into the game system of environmental regulations between local governments and enterprises. Some scholars have proposed that local governments’ behavioural decisions not only affect the improvement of regional environmental quality but also influence the environmental quality of neighbouring areas [9]. When the local region increases investment in environmental governance, the environmental quality of both the local and neighbouring regions will be improved. When the local region increases investment in production, pollutants will be generated, and pollutant spillover will lead to environmental damage in the neighbouring regions. In the context of fiscal decentralization, local governments have the characteristics of the ‘economic man’ and have more freedom and motivation to allocate financial funds according to their own will in terms of performance championship. How the spillover effect of local governments’ investment behaviours affects the choice of local governments’ environmental regulation execution strategy and enterprises’ green technology innovation strategy is the core issue of this section.

In view of this, based on the positive and negative external environmental effects of local governments’ investment behaviours, this paper establishes an evolutionary game model between local governments and enterprises to identify how spillover effects influence the implementation mechanism of environmental regulations. The evolutionary trajectories of local governments and enterprises to realize the system’s evolutionary stability equilibrium strategy under different circumstances are analysed. Furthermore, through numerical simulation, the influence of positive and negative externality spillover effects of local governments’ investment behaviours, punishment and reward for enterprises’ green technology innovation, and enterprises’ emission reduction capacity on the strategy choice of local governments and enterprises is further examined. The main contribution of this paper is that the spillover effect of local governments’ investment behaviour is incorporated into the study of the game relationship between local governments and enterprises, and the influence of the spillover effect on the strategy choice of local governments and enterprises is analysed. The existing literature focuses on the influence of incentive and constraint mechanisms among different stakeholders on the strategy choice of environmental regulatory subjects, while the spillover effect of local governments’ investment behaviour has not been considered. In addition, this paper adopts the method of case analysis combined with numerical simulation based on the research objects of the governments of Hubei Province, Hunan Province, and the YT Environmental Protection Technology Company, and quantitatively explores the impact of some key factors on the strategy choice of both parties in the game to effectively control the influence of the spillover effect. The conclusion of this paper provides a new explanation for the failure of environmental regulations and is conducive to identifying factors that affect the implementation distortion of environmental regulations, which provides a policy basis for the central government to improve local governments’ environmental regulation efficiency.

## 2. Literature Review

### 2.1. Study of the Game Relationship between Local Governments and Enterprises

The implementation process of environmental regulations is the game process of local governments and enterprises [10]. The academic research on the implementation mechanism of environmental regulations between local governments and enterprises mainly focuses on the following aspects: (1) the influence of environmental regulations on the strategic choice of the two players in the game. Zhi et al. [11] built an evolutionary game model between local governments and enterprises through an emission reduction subsidy policy. They found that increasing the disguised cost and expected risk cost of enterprises is conducive to promoting the government-enterprise game to achieve the ideal equilibrium. Yuan and Shi [12] used evolutionary game theory to study the interactions between government and enterprises and found that the incentives of government regulations, regulatory costs, and government subsidies were the main factors affecting the equilibrium outcomes of the evolutionary system. Yuan et al. [13] used the dynamic, time-given game model to analyse the equilibrium state and formation mechanism of the three-party game between the government, manufacturers, and retailers in different periods and stages and further compared and scrutinized the influence of the cooperative emission reduction mode and independent emission reduction mode on enterprises’ low-carbon technology innovation behaviour. (2) The influence of the third party on the strategic choice of both players. Sheng et al. [14] introduced third-party regulatory subjects and discussed how to effectively suppress collusion between government and enterprises. Chong and Sun et al. [15] also constructed a dual game model between the central government, local government, and enterprises and studied the impact of the reward and punishment mechanism of the central government on local government behaviour strategies. Xu [16] added public participation into an evolutionary game model of local governments and enterprises and concluded that improving public participation is helpful to realize the ideal equilibrium situation. Xiao [17] constructed an environmental regulation policy evolutionary game model of local governments and upstream and downstream enterprises and discussed hybrid environmental regulations regarding how to effectively promote the formation of the downstream supply chain.

### 2.2. Local Government Competition and Environmental Regulation Research

In the context of fiscal decentralization, performance appraisals based on GDP make local governments compete fiercely for resources to increase the probability of political promotion, thus affecting the impact of the implementation of environmental regulations. Due to the inevitable preference conflict between performance appraisals and residents’ welfare, local governments distort the implementation of environmental regulations [18]. Fiscal decentralization provides two conditions for local government competition, including financial and administrative power, and the performance evaluation index provides targets for local government competition [19]. Existing scholars have reached three inconsistent conclusions about the impact of local government competition on environmental regulations.

(1) The environmental regulation strategy of the ‘race to the bottom’

Most early scholars adopted the viewpoint of the ‘race to the bottom’; that is, local government competition, reduces the intensity of environmental regulations. Wilson [20] and Rauscher [21] believed that the existence of government competition forced local governments to relax environmental supervision and to reduce tax burdens and public spending, resulting in serious insufficient environmental input and aggravating environmental pollution. Zhang et al. [22] studied the behavioural characteristics of local governments against the background of ‘political centralization and fiscal decentralization’ and found that local governments compete with each other in environmental policy choice to obtain more liquidity factors. Zhou [23] revealed that the promotion tournament centred on ‘economic growth’ prompted local governments to pursue economic expansion, ignoring environmental governance. Shen et al. [24] further discovered that the investment behaviour of local governments in pursuit of economic growth has squeezed the input of environmental protection resources; at the same time, local officials have lowered the discharge standard to provide enterprises with more room for economic development, which has exacerbated environmental pollution.

(2) The environmental regulation strategy of the ‘race to the top’

With the deepening of the research, an increasing number of people put forward the view of the ‘race to the top’; that is, local government competition prompts local governments to increase the intensity of environmental regulations. Potoski [25] examined the interaction behaviours of state officials related to environmental regulations in the United States and found that the intensity of environmental regulations in each state showed an overall increasing trend. Holzinger and Sommerer [26] explored the behavioural characteristics of environmental policy formulation in more than 20 European countries and revealed the phenomenon of the ‘race to the top’. Zhang et al. [27] took the environmental regulation competition behaviour of local governments in various provinces of China as the research object and found that local governments’ competitive strategies were heterogeneous for different types of pollutants. Local governments had a ‘competition to the top’ strategy in CO_2_ emissions reduction but did not have competitive behaviour in SO_2_.

(3) The differentiated competition environmental regulation strategy

A few scholars have noted that environmental regulation competition among local governments involves differentiated competition; that is, local government competition has no significant impact on regional environmental policies. Chirinko and Wilson [28] showed that local governments adopt different competitive strategies for different types of environmental pollutant. Zhang et al. [29] explored the evolutionary features of environmental regulation intensity over time in interprovincial environmental regulation competition in China and demonstrated great differences in environmental regulation competition strategies among various provinces from 1998 to 2002; the gap in regulation intensity exhibited an expanding trend. Li and Luo [30] found that local governments adopt different competition strategies for different types of taxes, such as ‘optimal competition’ for macro tax burdens, ‘inferior competition’ for corporate income tax, and ‘seesaw riding’ for value-added and environmental taxes.

### 2.3. The Spillover Effect of Local Governments’ Investment Behaviour

In our research, local governments’ behaviour refers to the supporting behaviours and preferential policies related to economic development and environmental protection. Competition among local governments causes officials in different regions to imitate or fight over policies around competition for promotion opportunities, the supply of public products, and the introduction of liquidity factors, which aggravates the spillover effect of local governments’ investment behaviour on regional development. There are many types of formal and informal connection in regional economic activities, which makes it easier for enterprises in the region to obtain information and knowledge from neighbouring regions. Therefore, the decision-making behaviour of regional local governments has mutual influence and mutual radiation, leading to a positive spillover effect and a negative spillover effect.

On the one hand, the behaviour of local governments supporting local economic development has a negative externality on the environmental governance of adjacent areas, which has increased environmental pollution due to the improvement of regional production capacity. Hao et al. [31] found that local governments will use more resources to support economic construction when they experience corruption, and the spatial spillover effect of a large amount of ‘three industrial wastes’ produced by local industrial enterprises has exacerbated environmental pollution in neighbouring areas. Sun et al. [32] also pointed out that local governments’ policies to support local economic construction have led to the transfer of pollutants across regions and increased environmental governance costs in neighbouring areas. As a result, neighbouring governments adopt the same preferential economic policies and neglect environmental protection, thus forming a ‘tragedy of the commons’. Ijiri and Jinushi [33] focused on the policy spillover effects between different countries and found that the quantitative easing of a policy’s international spillover by the United States has a significant impact on the development of Japan’s real economy and financial market. In addition, the spillover effect has a greater impact on the real economy in the early stage, while it has a greater impact on the financial market in the later stage.

On the other hand, the behaviour of local governments supporting local environmental governance has improved the local environmental quality due to the reduction of environmental pollution and has a positive externality to the environmental governance of neighbouring areas. First, regional environmental regulation has a positive environmental spillover effect, which comes from the regional ‘demonstration effect’ and ‘warning effect’. Zhao et al. [34] found that strict supervision has a deterrent effect on enterprises in neighbouring regions with high economic levels, resulting in imitation behaviour and promoting green technology diffusion. Yu and Li [35] verified the spillover effect of China’s carbon emissions trading policy through empirical analysis and found that the governments of surrounding areas choose the same environmental regulation policies as the carbon trading pilot provinces and cities, which produces a synergistic effect on the environmental governance of the surrounding provinces.

The spillover effect can occur horizontally or vertically, so the measure of spillover effects in the existing literature varies with the direction of spillover. In the study of vertical spillover effects, Newman et al. [36] used the growth rate of the proportion of similar product income of foreign-funded enterprises in the income of upstream enterprises as the measure of the spillover effect. Prtr and Jan [37] studied the spillover effects between enterprises and external entities such as the market, universities and scientific research institutions, which were approximated by evaluating the importance of chosen communication sources according to a knowledge spillover model. In a study of horizontal spillover effects, Javorcik et al. [38] used the growth rate of the proportion of foreign-funded enterprises’ income in the total income of the industry to capture the horizontal spillover effect. Djulius et al. [39] used the ratio of the number of workers in foreign companies to the number of workers in domestic companies to measure the knowledge spillover effect caused by foreign investment. Aldieri et al. [40] studied the impact of knowledge spillovers in innovative activities on employment in Finland and used the cross-effect of regional R&D capital stock and geographic distance to measure the effect of knowledge spillovers.

By combining and summarizing the existing literature, we can see that existing scholars have conducted considerable research on local government competition and environmental regulations. It is widely believed that local government competition has a significant impact on regional environmental protection enforcement investment, but there are great differences in the research conclusions. In addition to differences in index measurement, research methods and research sample selection, the key problem is the lack of in-depth research on environmental regulation transmission mechanisms, ignoring the spillover effect of local governments’ behaviour. In fact, the strategy choice of local governments competing with each other is the reaction function of each other’s behavioural decisions. When the local area increases the investment in environmental protection, the environmental quality and the neighbouring areas will also be improved. When the local area increases the investment in production, pollutant spillover will cause environmental damage to neighbouring areas.

Yi [41] established the local governments’ environmental protection investment game model, and concluded that the spillover of local governments’ investment in water environment governance is the main cause of the insufficient investment in water pollution governance. Referring to the research of Yi, the spillover effect of local governments’ investment behaviour on environmental regulation subject strategy choice is analysed to improve the environmental policy implementation mechanism and to provide theoretical guidance for the central government to formulate a sound green technology innovation supervision system.

## 3. Model Construction and Analysis

### 3.1. Problem Description and Assumptions

In China’s administrative system, the central government is responsible for formulating environmental policies, while local governments supervise and manage enterprises’ energy conservation and emission reduction activities by implementing environmental regulations. Local governments are responsible for the economic construction and environmental protection of the region, so local governments have two types of investment behaviour. The first is economic constructive investment, which refers to local governments providing financial support for local natural resource development and infrastructure construction to encourage enterprise to actively participate in activities related to economic development. The second is environmental protection investment, which refers to local governments providing financial support to promote enterprises to conduct energy conservation and emission reduction, including special funds, energy conservation and emission reduction subsidies, and tax incentives. The behaviour of local governments in supporting local economic development has a negative externality on the environmental governance of adjacent areas, which has increased environmental pollution due to the improvement of regional production capacity. The behaviour of local governments in supporting local environmental governance has improved the local environmental quality due to the reduction of environmental pollution and has a positive externality to the environmental governance of neighbouring areas.

To simplify the analysis, this paper takes market incentive environmental regulation as an example. Local governments will charge enterprises to discharge pollutants to keep their emissions within a certain range. In addition, local governments’ environmental protection investment behaviour is affected by the spillover effect of neighbouring governments’ investment behaviour.

**Assumption** **1.** *Based on the work of Yi [41], there are two geographically adjacent local governments of* a *and* b *within the jurisdiction of the central government. The total investment budget of the local government is* m *, which can be used to support the development of environmental protection and economic construction. Either party’s investment in environmental protection can bring benefits to both parties, any party’s investment in economic production can benefit itself, and the production of pollutants can reduce the benefits of the other party. Namely, the economic constructive investment* N *has a negative externality, and the environmental protection investment behaviour* P *has positive externalities.*

**Assumption** **2.** *Local governments’ environmental protection investment*P*not only brought environmental benefits*$h_{1}P$*to local governments but also contributed to reducing the cost of enterprise green technology innovation. The cost reduction of enterprises is*$h_{2}P$*, where*$h_{1}$*and*$h_{2}$*are the environmental benefit coefficients obtained by local governments and enterprises, respectively, and*$h_{1}>0,h_{2}>0$.

**Assumption** **3.** *If the local government strictly implements environmental regulations, the reward for an enterprise’s complete green technology innovation behaviour is* B *, and the punishment for an enterprise’s incomplete green technology innovation behaviour is* F *. At the same time, when the enterprise’s emission reduction rate is* l *, the local government charges pollutant discharge fees* $k(1-r_{2}l)$ *for the enterprise’s pollutant discharge, and* k *is the sewage charge that the enterprise needs to pay per unit of sewage discharge (* k>0 *).*

### 3.2. Model Building

Based on the literature of Yi [41], the investment benefit function of local government a and local government b is assumed to be in the form of a Cobb Douglas function:

(1) $$R_{a}={\left(P_{a}+uP_{b}\right)}^{\alpha }{\left(N_{a}-wN_{b}\right)}^{\beta }$$

where i,j=a,b and i≠j, 0<α,β,u,w<1;α+β<1, u is the positive externalities’ coefficient, w is the negative externalities’ coefficient, $P_{a}$ and $P_{b}$ represent the environmental protection investment of governments a and b. $N_{a}$ and $N_{b}$ denote the economic constructive investment of governments a and b. To maximize the interests of both sides, the two governments can adopt two strategies of cooperation and non-cooperation, and the investment intensity of environmental protection is different under different strategies:

When governments a and b compete with each other, both parties seek to maximize their personal interests to gain competitive advantages, namely:

(2) $$\begin{array}{l} \left\{\begin{array}{l} \max R_{a}={\left(P_{a}+uP_{b}\right)}^{\alpha }{\left(N_{a}-wN_{b}\right)}^{\beta }\\ P_{a}+N_{a}=m \end{array}\right.\\ \left\{\begin{array}{l} \max R_{b}={\left(P_{b}+uP_{a}\right)}^{\alpha }{\left(N_{b}-wN_{a}\right)}^{\beta }\\ P_{b}+N_{b}=m \end{array}\right. \end{array}$$

where m refers to the total investment budget of the local government, which can be used to support environmental protection and economic development.

Combined with optimization theory according to Formula (2), the optimal environmental protection investment benefit $P_{a}*$ and $P_{b}*$ can be calculated under the competitive situation as follows:

$$P_{a}*=P_{b}*=\frac{\alpha -\alpha w}{\beta +\beta u+\alpha -\alpha w}m$$

If the local government chooses the strategy of strict supervision and the enterprise chooses the strategy of complete green technology innovation, the local government’s profit function consists of environmental investment returns $h_{1}P$, regulatory costs $c_{1}$, investment in environmental protection P, and revenue from sewage charges k(1−l). The enterprise’s profit function consists of the environmental returns of local government investment $h_{2}P$, green technology innovation costs $c_{2}$, and sewage charges k(1−l). If the local government chooses the strategy of strict supervision and the enterprise chooses the strategy of incomplete green technology innovation, the local government’s profit function consists of environmental investment benefits $h_{1}P$, regulatory costs $c_{1}$, environmental protection investment costs P, and revenue from sewage charges $k(1-r_{2}l)$. The enterprise’s profit function consists of the environmental investment benefits of local governments $h_{2}P$, green technology innovation costs $r_{2}c_{2}$, and sewage charges $k(1-r_{2}l)$. If the local government chooses the strategy of non-strict supervision and the enterprise chooses the strategy of complete green technology innovation, the local government’s profit function consists of environmental investment benefits $r_{1}h_{1}P$, regulatory costs $r_{1}c_{1}$, environmental protection investment cost $r_{1}P$, and revenue from sewage charges $k(1-l)r_{1}$. The enterprise’s profit function consists of the environmental benefits of local government investment $r_{1}h_{2}P$, green technology innovation costs $r_{2}c_{2}$, and sewage charges $k(1-l)r_{1}$. If the local government chooses the strategy of non-strict supervision and the enterprise chooses the strategy of incomplete green technology innovation, the local government’s profit function consists of environmental investment benefits $r_{1}h_{1}P$, regulatory costs $r_{1}c_{1}$, environmental protection investment costs $r_{1}P$, and revenue from sewage charges $k(1-r_{2}l)r_{1}$. The enterprise’s profit function consists of the environmental benefits of local government investment $r_{1}h_{2}P$, green technology innovation costs $r_{2}c_{2}$, and sewage charges.

According to the above description, the game payment matrix of local governments and enterprises can be obtained, as shown in Table 1.

### 3.3. Dynamic Analysis of Evolutionary Strategy Replication

x is the probability of an enterprise choosing the strategy of complete green technology innovation. The probability of an enterprise choosing the strategy of incomplete green technology innovation is 1−x. y is the probability of the local government choosing the strategy of strict supervision. The probability of the local government choosing the strategy of non-strict supervision is 1−y.

An enterprise’s adaptability in choosing the strategy of complete green technology innovation is as follows:

(3) $$U_{1}=y[-k(1-l)+B+h_{2}P-c_{2}]+(1-y)[r_{1}B+r_{1}h_{2}P-k(1-l)r_{1}-c_{2}]$$

An enterprise’s adaptability in choosing the strategy of incomplete green technology innovation is as follows:

(4) $$U_{2}=y[h_{2}P-k(1-r_{2}l)-F-r_{2}c_{2}]+(1-y)[r_{1}h_{2}P-r_{2}c_{2}-k(1-r_{2}l)r_{1}-r_{1}F]$$

The average fitness is:

(5) $$\bar{U}=xU_{1}+(1-x)U_{2}$$

The dynamic equation of enterprise replication is as follows:

(6) $$\frac{dx}{dt}=y(U_{1}-\bar{U})=y(1-y)(U_{1}-U_{2})$$

The local government’s adaptability in choosing the strategy of strict supervision is:

(7) $$V_{1}=x[k(1-l)-B+(h_{1}-1)P-c_{1}]+(1-x)[k(1-r_{2}l)-c_{1}+(h_{1}-1)P+F]$$

The local government’s adaptability in choosing the strategy of non-strict supervision is:

(8) $$V_{2}=x[k(1-l)r_{1}-r_{1}B+r_{1}(h_{1}-1)P-r_{1}c_{1}]+(1-x)[k(1-r_{2}l)r_{1}-r_{1}c_{1}+r_{1}(h_{1}-1)P+r_{1}F]$$

The average fitness is:

(9) $$\bar{V}=yV_{1}+(1-y)V_{2}$$

Likewise, the replication dynamic equation of the local government is as follows:

(10) $$\frac{dy}{dt}=y(V_{1}-\bar{V})=y(1-y)(V_{1}-V_{2})$$

According to the Malthusian equation [42], the growth rate of strategy probability is equal to its fitness minus the average fitness, and a two-dimensional dynamic system of local government and enterprise can be obtained:

(11) $$\left\{\begin{array}{cc} \frac{dx}{dt} & =x(1-x)\{(klr_{1}-c_{2})(1-r_{2})+(B+F)r_{1}+(1-r_{1})[kl(1-r_{2})+B+F]y\}\\ \frac{dy}{dt} & =y(1-y)(1-r_{1})\{k(1-r_{2}l)+F-C_{1}+(h_{1}-1)P+[kl(r_{2}-1)-B-F]x\} \end{array}\right.$$

The strategy combination, corresponding to the equilibrium point calculated by Equation (11), is an equilibrium of the evolutionary game.

**Proposition** **1.** *The equilibrium point of system (11) is (0, 0)(0, 1)(1, 0)(1, 1),*

*When:*

$$\left\{\begin{array}{c} 1>\frac{k(1-r_{2}l)+F-C_{1}+(h_{1}-1)P}{kl(1-r_{2})+B+F}>0\\ 1>\frac{(c_{2}-klr_{1})(1-r_{2})-(B+F)r_{1}}{\left(1-r_{1})[kl(1-r_{2})+B+F\right]}>0 \end{array}\right.$$

$$x*=\frac{k(1-r_{2}l)+F-C_{1}+(h_{1}-1)P}{kl(1-r_{2})+B+F}$$

$$y*=\frac{(c_{2}-klr_{1})(1-r_{2})-(B+F)r_{1}}{\left(1-r_{1})[kl(1-r_{2})+B+F\right]}$$

*so the system’s equilibrium point is*(x*, y*).

**Proof.**  According to equations $\frac{dx}{dt}=0$ and $\frac{dy}{dt}=0$ x*, y* can be obtained as (0, 0), (0, 1), (1, 0), (1, 1), and (x*, y*). □

Set $A_{1}=(1-r_{2})(kl-c_{2})+B+F$, which represents the difference between the enterprise’s return to choosing the strategy of complete green technology innovation and choosing the strategy of incomplete green technology innovation when the local government selects the strategy of strict supervision. Set $A_{2}=(1-r_{1})[c_{1}+(1-h_{1})P-k(1-r_{2}l)-F]$, which denotes the difference between the local government’s return to choosing the strategy of non-strict or strict supervision when the enterprise chooses the strategy of incomplete green technology innovation. Set $A_{3}=(1-r_{2})(c_{2}-klr_{1})-r_{1}B-r_{1}F$, which represents the difference between the enterprise’s return to choosing the strategy of incomplete or complete green technology innovation when the local government chooses the strategy of non-strict supervision. Set $A_{4}=(1-r_{1})[k(1-l)-c_{1}+(h_{1}-1)P-B]$, which represents the difference between the local government’s return of choosing the strategy of strict or non-strict supervision when the enterprise chooses the strategy of complete green technology innovation. According to the expressions of $A_{1}$, $A_{2}$, $A_{3}$, $A_{4}$ it can be found that $A_{1}>-A_{3},A_{4}<-A_{2}$.

According to Equation (11), the Jacobian matrix of the system can be obtained as follows A=∂x*x∂x*y∂y*x∂y*y=a11a12a21a22, where:

$$a_{11}=(1-2x)\{(klr_{1}-c_{2})(1-r_{2})+(B+F)r_{1}+(1-r_{1})[kl(1-r_{2})+B+F]y\}$$

$$a_{12}=x(1-x)(1-r_{1})[B+F+(1-r_{2})kl]$$

$$a_{21}=y(1-y)[(r_{2}-1)(1-r_{1})kl-B-F]$$

$$a_{22}=(1-2y)(1-r_{1})\{k(1-r_{2}l)+F-C_{1}+(h_{1}-1)P+[kl(r_{2}-1)-B-F]x\}$$

Substituting the equilibrium point into equations $trJ=a_{11}+a_{22}$ and $\det J=\left|\left.\begin{array}{cc} a_{11} & a_{12}\\ a_{21} & a_{22} \end{array}\right|\right.$, the expression of determinant det(A) and trace tr(A) related to the four equilibrium points can be obtained, as shown in Table 2.

### 3.4. Stability Analysis of Evolutionary Strategy

**Figure 1:**
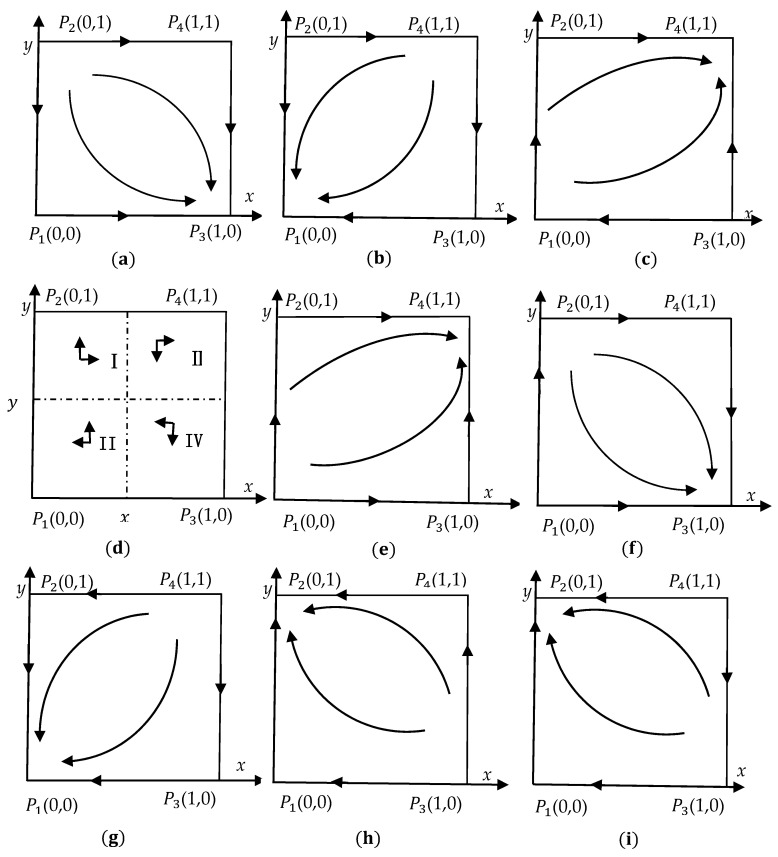
Dynamic phase diagram of system evolution under nine situations. **Note:** Figures (**a**–**i**) refers to the figure number of dynamic phase diagram of system evolution under situation 1–9.

When $\left\{\begin{array}{c} tr(A)=a_{11}+a_{22}<0\\ \det(A)=\left|\begin{array}{cc} a_{11} & a_{12}\\ a_{21} & a_{22} \end{array}\right|>0 \end{array}\right.$, the equilibrium point of the replication dynamic equation is an evolutionarily stable strategy [43]. Combined with det(A), tr(A) and the system phase diagram, the stability of equilibrium points under 9 situations is analysed, as shown in Table 3, Table 4 and Table 5 and Figure 1.

Situation 1: When $A_{1}>0,A_{2}>0,A_{3}<0,A_{4}<0$, no matter what strategy the local government chooses, the enterprise’s benefit of choosing complete green technology innovation is greater than that of choosing incomplete green technology innovation. Regardless of the strategy the enterprise chooses, the local government’s benefit of choosing the strategy of non-strict supervision is greater than that of choosing strict supervision. The local equilibrium point is shown in Table 3, and the dynamic evolutionary process of the two parties in the game is shown in Figure 1a. The point of (1, 0) is the evolutionary stability strategy (ESS). This equilibrium situation stems from the fact that when the cost of the local government’s strict supervision is relatively high and the cost of the enterprise’s complete green technology innovation is relatively low, both the local government and the enterprise are rational economic actors who pursue their own interests for maximization and tend to choose the most favourable strategy (strict supervision, complete green technology innovation).

Situation 2: When $A_{1}>0,A_{2}>0,A_{3}>0,A_{4}<0$, the profit of an enterprise choosing complete green technology innovation is greater than that of an enterprise choosing incomplete green technology innovation when the local government chooses strict supervision. The profit of an enterprise choosing incomplete green technology innovation is greater than that of an enterprise choosing complete green technology innovation when the local government chooses non-strict supervision. Regardless of the strategy the enterprise chooses, the local government’s benefit of choosing the strategy of non-strict supervision is greater than that of choosing strict supervision. The local equilibrium is shown in Table 3, and the dynamic evolutionary process of the two parties in the game is displayed in Figure 1b. The point of (0, 0) is the ESS. This means that the high cost of the local government choosing strict supervision leads to its net profit in choosing strict supervision being lower than that of a local government choosing non-strict supervision. To maximize the local government’s own interests, choosing non-strict supervision is the best strategy and reduces the risk cost of an enterprise’s incomplete green technology innovation. The high cost of an enterprise choosing green technology innovation means that the net profit of an enterprise choosing incomplete green technology innovation is greater than that of an enterprise choosing complete green technology innovation. To maximize the enterprise’s own interests, incomplete green technology innovation is the best choice.

Situation 3: When $A_{1}>0,A_{2}<0,A_{3}>0,A_{4}>0$, the profit of an enterprise choosing complete green technology innovation is greater than that of an enterprise choosing incomplete green technology innovation when the local government chooses strict supervision. The profit of an enterprise choosing incomplete green technology innovation is greater than that of an enterprise choosing complete green technology innovation when the local government chooses non-strict supervision. Regardless of the strategy the enterprise chooses, the local government’s benefit of choosing the strategy of strict supervision is greater than that of choosing non-strict supervision. The local equilibrium is shown in Table 3, and the dynamic evolutionary process of the two parties in the game is portrayed in Figure 1c. The point of (1, 1) is the ESS. This means that the high benefit of the local government in choosing strict supervision leads to the net profit of the local government in choosing strict supervision being greater than that of a local government choosing non-strict supervision. To maximize the local government’s own interests, choosing strict supervision is the best strategy; it increases the risk cost of an enterprise’s incomplete green technology innovation and leads to the enterprise’s net profit of choosing complete green technology innovation being greater than that of an enterprise choosing incomplete green technology innovation. Complete green technology innovation is the best choice.

Situation 4: When $A_{1}>0,A_{2}<0,A_{3}>0,A_{4}<0$, the profit of an enterprise choosing complete green technology innovation is greater than that of an enterprise choosing incomplete green technology innovation when the local government chooses strict supervision. Meanwhile, the profit of an enterprise choosing incomplete green technology innovation is greater than that of an enterprise choosing complete green technology innovation when the local government chooses non-strict supervision. The profit of a local government choosing the strategy of strict supervision is greater than that of choosing non-strict supervision when the enterprise chooses the strategy of incomplete green technology innovation. The profit of a local government’s benefit in choosing the strategy of non-strict supervision is greater than that of choosing strict supervision when the enterprise chooses the strategy of complete green technology innovation. The local equilibrium is depicted in Table 4, and the dynamic evolutionary process of the two parties in the game is outlined in Figure 1d.

According to the equation of

$$F(x)=\frac{dx}{dt}=x(1-x)\{(klr_{1}-c_{2})(1-r_{2})+(B+F)r_{1}+(1-r_{1})[kl(1-r_{2})+B+F]y\}=0$$

and x=0 x=1. If $y>\frac{(c_{2}-klr_{1})(1-r_{2})-(B+F)r_{1}}{\left(1-r_{1})[kl(1-r_{2})+B+F\right]}$, F(x)>0, $F^{\prime }(0)>0$, and $F^{\prime }(1)<0$, then the steady state is x=1. If $y<\frac{(c_{2}-klr_{1})(1-r_{2})-(B+F)r_{1}}{\left(1-r_{1})[kl(1-r_{2})+B+F\right]}$, so F(x)<0, $F^{\prime }(0)<0$, $F^{\prime }(1)>0$, then the steady state is x=0. According to the equation of

$$F(y)=\frac{dy}{dt}=y(1-y)(1-r_{1})\{k(1-r_{2}l)+F-C_{1}-P+[kl(r_{2}-1)-B-F]x\}=0$$

the steady state is y=0 and y=1. If $x<\frac{k(1-r_{2}l)+F-C_{1}+(h_{1}-1)P}{kl(1-r_{2})+B+F}$, the steady state is y=1. If $x>\frac{k(1-r_{2}l)+F-C_{1}+(h_{1}-1)P}{kl(1-r_{2})+B+F}$, the steady state is y=0. The critical values of $x*=\frac{k(1-r_{2}l)+F-C_{1}+(h_{1}-1)P}{kl(1-r_{2})+B+F}$ and $y*=\frac{(c_{2}-klr_{1})(1-r_{2})-(B+F)r_{1}}{\left(1-r_{1})[kl(1-r_{2})+B+F\right]}$ divide the evolutionary game phase diagram into four regions: I, II, III, and IV.

When the initial state of the system falls in region I, the game converges to (1, 1). When the initial state of the system falls in region II, the game converges to (1, 0). When the initial state of the system falls in region III, the game converges to (0, 1). When the initial state of the system falls in region IV, the game converges to (0, 0).

Situation 5: When $A_{1}>0,A_{2}<0,A_{3}<0,A_{4}>0$, no matter what strategy the local government chooses, the profit of an enterprise choosing complete green technology innovation is greater than that of choosing incomplete green technology innovation. Regardless of the strategy the enterprise chooses, the profit of the local government in choosing the strategy of strict supervision is greater than that of choosing non-strict supervision. The local equilibrium is shown in Table 4, and the dynamic evolutionary process of the two parties in the game is displayed in Figure 1f. The point of (1, 1) is the ESS. The relatively low cost of strict supervision leads to the net profit of the local government choosing strategy of strict supervision being greater than that of choosing non-strict supervision, so the strategy of strict supervision is the best choice. The relatively low cost of complete green technology innovation means that the profit of an enterprise in choosing complete green technology innovation is greater than that of an enterprise choosing incomplete green technology innovation, so the strategy of complete green technology innovation is the best choice.

Situation 6: When $A_{1}>0,A_{2}<0,A_{3}<0,A_{4}<0$, no matter what strategy the local government chooses, the profit of an enterprise in choosing complete green technology innovation is greater than that of choosing incomplete green technology innovation. The profit of the local government in choosing the strategy of strict supervision is greater than that of choosing non-strict supervision when the enterprise chooses the strategy of incomplete green technology innovation. The profit of the local government in choosing the strategy of non-strict supervision is greater than that of choosing strict supervision when the enterprise chooses the strategy of complete green technology innovation. The local equilibrium is portrayed in Table 4, and the dynamic evolutionary process of the two parties in the game is shown in Figure 1g. The point of (1, 0) is the ESS. This means that the relatively low cost of complete green technology innovation indicates that the profit of an enterprise in choosing complete green technology innovation is greater than that of an enterprise choosing incomplete green technology innovation, so the strategy of complete green technology innovation is the best choice. To reduce the cost of regulation, non-strict supervision is the best choice for local governments.

Situation 7: When $A_{1}<0,A_{2}>0,A_{3}>0,A_{4}<0$, no matter what strategy the local government chooses, the profit of an enterprise in choosing incomplete green technology innovation is greater than that of an enterprise choosing complete green technology innovation. Regardless of the strategy the enterprise chooses, the profit of the local government in choosing the strategy of non-strict supervision is greater than that of choosing strict supervision. The local equilibrium is shown in Table 5, and the dynamic evolutionary process of the two parties in the game is outlined in Figure 1h. The point of (0, 0) is the ESS. This means that when the cost of strict supervision is high, the strategy of non-strict supervision is the best choice for the local government. When the cost of incomplete green technology innovation is low, the strategy of incomplete green technology innovation is the best choice for an enterprise.

Situation 8: When $A_{1}<0,A_{2}<0,A_{3}>0,A_{4}>0$, no matter what strategy the local government chooses, the profit of an enterprise in choosing incomplete green technology innovation is greater than that of an enterprise choosing complete green technology innovation. Regardless of the strategy the enterprise chooses, the profit of the local government in choosing the strategy of strict supervision is greater than that of choosing non-strict supervision. The local equilibrium is shown in Table 5, and the dynamic evolutionary process of the two parties in the game is displayed in Figure 1h. The point of (0, 1) is the ESS. The high profit of strict supervision makes the local government more likely to strictly implement environmental regulation. The low cost of incomplete green technology innovation makes the enterprise more likely to give up on a strategy of complete green technology innovation.

Situation 9: When $A_{1}<0,A_{2}<0,A_{3}>0,A_{4}<0$, no matter what strategy the local government chooses, the profit of an enterprise in choosing incomplete green technology innovation is greater than that of an enterprise in choosing complete green technology innovation. The profit of the local government in choosing the strategy of strict supervision is greater than that of choosing non-strict supervision when the enterprise chooses the strategy of incomplete green technology innovation. The profit of the local government in choosing the strategy of non-strict supervision is greater than that of choosing strict supervision when the enterprise chooses the strategy of complete green technology innovation. The local equilibrium is depicted in Table 5, and the dynamic evolutionary process of the two parties in the game is shown in Figure 1i. The point of (0, 1) is the ESS. This can be explained by the fact that the low profit of complete green technology innovation makes the enterprise more likely to give up on the strategy of complete green technology innovation. In addition, the loss of the local government in choosing the strategy of strict supervision is lower than the loss of choosing the strategy of non-strict supervision, so the strategy of strict supervision is the best choice.

## 4. Case Analysis and Numerical Simulation

### 4.1. The Key Factors Affecting the Evolutionary Path

Based on the above dynamic evolutionary model, there are four evolutionary stability strategies for the behavioural choice of local governments and enterprises in the environmental regulation transmission mechanism. In addition, the evolutionary path of the two parties in the game is related to the game payout matrix and changes in some central parameters. The impact of the main factors related to the behavioural characteristics of local governments and enterprises on the equilibrium results of system evolution has been discussed.

(1) The impact of the spillover of local governments’ investment behaviour on the choice of local governments’ environmental regulation strategy.

The spillover of local governments’ investment behaviour reflects the impact of regional government environmental regulation decisions on the environmental performance of neighbouring regions. Whether the spillover effect is positive or negative related to the probability of local governments choosing the strategy of strict supervision depends on whether the spillover effect is a ‘demonstration effect’ or a ‘free rider’ on the neighbouring areas. When the environmental protection awareness of local residents is low, environmental regulations cannot effectively encourage local enterprises to consciously save energy and reduce emissions, and the environmental protection investment benefits of local governments are relatively low. Therefore, neighbouring regional governments and enterprises have ‘free-riding’ behaviours in energy conservation and emission reduction. Local governments will be more likely to choose strict supervision strategies because of the low enthusiasm of local enterprises to save energy and reduce emissions. When the environmental protection awareness of local residents is high, environmental regulations can effectively encourage local enterprises to consciously save energy and reduce emissions, and the environmental protection investment performance of local governments is relatively large. Hence, the environmental quality improvement and environmental pollution brought by the investment of local governments have a ‘warning effect’ and ‘demonstration effect’ on the environmental protection of neighbouring areas. Local governments will be more likely to abandon strict supervision strategies because of the high enthusiasm of local enterprises to save energy and reduce emissions.

**Proof.**  Please see the Proof of Proposition A1 in Appendix A. □

(2) The impact of local government reward and punishment for enterprises’ green technology innovation behaviours on the strategy choice of local governments and enterprises.

The punishment and reward for enterprises’ green technology innovation behaviour by the local government is directly related to the costs and benefits of enterprises and local governments. The higher the reward for enterprises’ complete green technology innovation behaviour, the greater the green technology innovation benefits of the enterprises, and the higher the environmental protection cost of the local government. The greater the punishment for enterprises’ incomplete green technology innovation behaviour, the higher the green technology innovation cost of enterprises, and the lower the environmental protection cost of local governments. Therefore, the greater the reward, the greater the probability of enterprises choosing complete green technology innovation strategy to obtain more benefits, and the lower the probability of local governments choosing the strict regulation strategy to reduce management costs. The greater the punishment is, the greater the probability of enterprises choosing a complete green technology innovation strategy to reduce costs. The impact of punishment on the choice of the local government’s environmental regulation strategy depends on the trade-off between the environmental benefits and economic losses brought about by the punishment.

**Proof.**  Please see the Proof of Proposition A2 in Appendix A. □

(3) The impact of enterprises’ emission reduction capability on the strategy choice of local governments and enterprises.

Emission reduction capability determines the difficulty of green technology innovation for enterprises. The stronger the emission reduction capability is, the more easily enterprises can develop and utilize clean technologies, and the higher the willingness of enterprises to choose complete green technology innovation. The high willingness of enterprises to engage in green technology innovation reduces the effectiveness of strict environmental supervision, and the local government will abandon the strategy of strict environmental supervision.

**Proof.**  Please see the Proof of Proposition A3 in Appendix A. □

### 4.2. Numerical Simulation Analysis

To better describe the influence of various parameters on the strategy choice of local governments and enterprises in the transmission mechanism of environmental regulations, a combination of case analysis and numerical simulation has been adopted to quantitatively examine the influence of different parameters on the choice behaviour of both sides of the game.

The 11 provinces of the Yangtze River Economic Belt regard the restoration of the Yangtze River’s ecological environment as an overwhelming task. Lake governance, especially the governance of cross regional large lakes, requires the joint efforts of all governments in the basin, realizing joint prevention and control. If only one party carries out pollution control and the other party carries out pollution discharge, it is difficult to achieve the expected effect of environmental improvement. Hunan and Hubei provinces have similar economic development and adjacent geographic positions in the central region and are two important areas adjacent to the Yangtze River Economic Belt, the ecological restoration of which plays an important role in the growth of the Yangtze River Economic Belt. Although the central government and provincial government have invested a lot of funds in lake protection and governance, the efficiency of governance is low due to the problems of cross regional sewage transfer and unclear responsibility for pollution control. Therefore, grasping the spillover effects of local government investment behavior is a prerequisite for establishing an effective joint prevention and control mechanism in the governance of trans-basin rivers.

As an important environmental protection enterprise in the Yangtze River Economic Zone, YT Environmental Protection Technology Co., Ltd. is a company specializing in environmental engineering general contracting, environmental protection facility renovation, trusteeship operation, water treatment pharmaceutical series products, and environmental protection equipment research and development. Its green technology innovation behaviour is supervised and managed by the Hunan provincial government. This paper takes the Hubei provincial government, Hunan provincial government, and YT Environmental Protection Technology Co., Ltd. as the research objects and focuses on the influence of the competition between the Hubei provincial government and the Hunan provincial government on the choice of Y T Environmental Protection Technology Co., Ltd.’s green technology innovation strategy.

For the sake of evolutionary game analysis, the key stakeholders are simplified as Hunan province local government and the YT company, focusing on the behavioral spillover effect of the Hubei province local government. Based on in-person investigations and data collection from the relevant government departments and enterprises, we use the following series of parameter values as the benchmark: the supervision intensity of local governments choosing strategy of no-strict supervision as $r_{1}=0.3$, the effort of enterprise choosing strategy of incomplete green technology innovation $r_{2}=0.2$, the sewage charge k=2, the punishment for the enterprise’s incomplete green technology innovation behaviour F=5, the reward for the enterprise’s complete green technology innovation behaviour B=4, regulatory costs of local government $C_{1}=1$, the green technology innovation costs of enterprise $C_{2}=2$, the environmental benefit coefficients obtained by the Hunan provincial local government and the YT enterprise from the local government’s environmental protection investment $h_{1}=h_{2}=0.3$, the positive externalities’ coefficient and the negative externalities’ coefficient u=w=0.3. While a constellation of additional parameters is held at: α=0.2,β=0.3.

**Figure 2:**
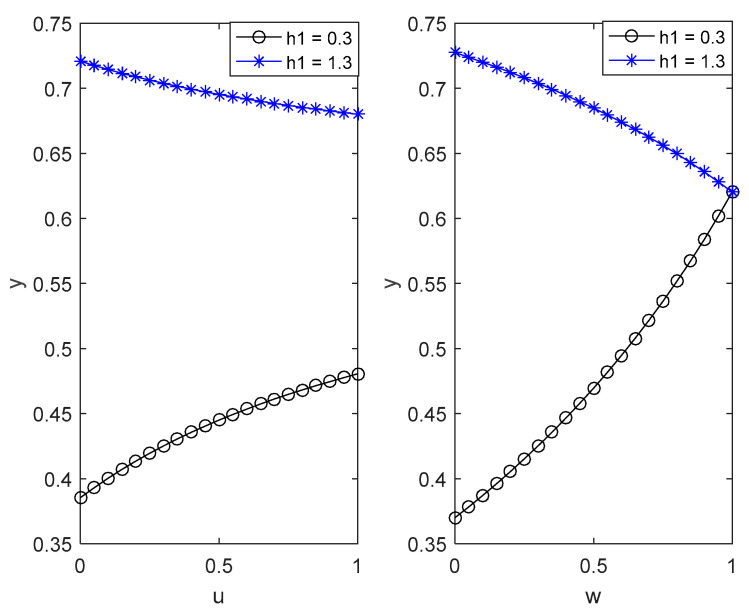
The influence of the external government investment behaviour externality coefficient on local government environmental supervision behaviour.

**Figure 3:**
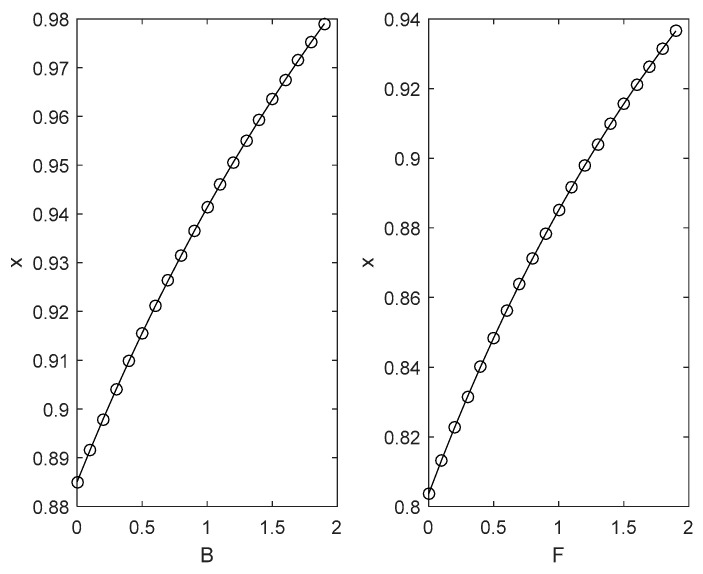
The impact of reward B and punishment F on enterprises’ green technology innovation behaviour.

**Figure 4:**
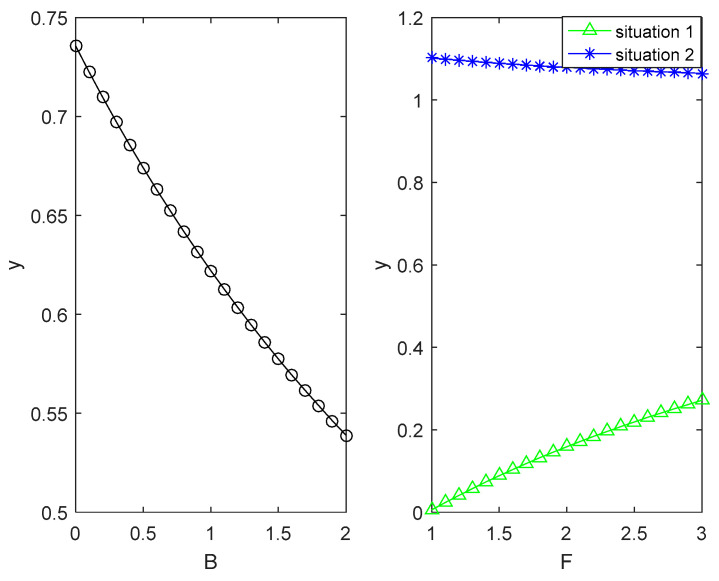
The impact of reward B and punishment F on local governments’ environmental regulation strategy supervision behaviour.

**Figure 5:**
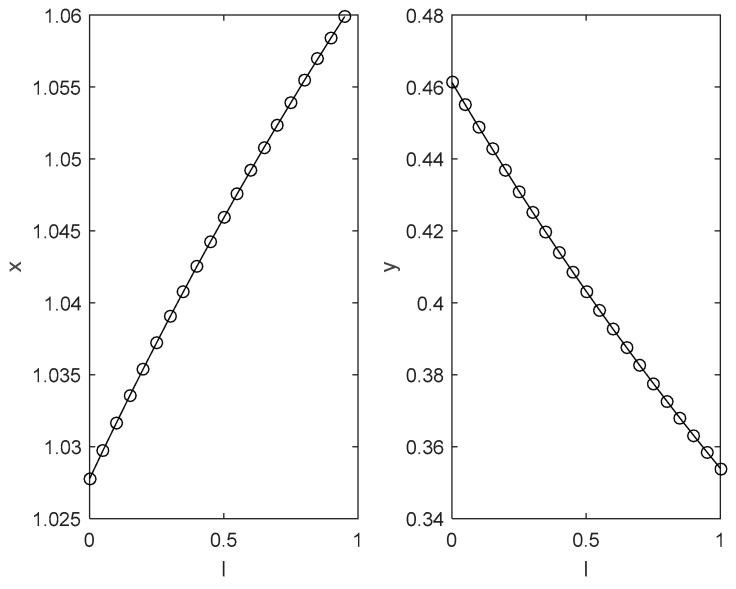
The impact of enterprises’ emission reduction capacity on the choice of enterprises’ green technology innovation strategy and local governments’ environmental regulation strategy.

According to the benchmark value, the influence of different key parameters on the evolutionary trajectory of the two sides of the game is numerically simulated and analysed as shown in Figure 2, Figure 3, Figure 4 and Figure 5.

(1) Figure 2 shows the impact of the positive externality coefficient u and negative externality coefficient w of external government investment behaviour on the probability of the local government choosing strict supervision strategies in two cases under the condition that other parameters remain unchanged.
  (i) As seen in Figure 2, when $h_{1}=0.3$, it meets the condition that $h_{1}-1<0$; with the increase of the positive externality coefficient u and the negative externality coefficient w, the probability of the local government choosing strict supervision strategies will increase rapidly. However, the rising speed under the influence of positive externality coefficients continues to decline, and the rising speed under the influence of negative externality coefficients continues to accelerate. In practice, low environmental protection benefits mean that environmental policies cannot effectively motivate local enterprises to consciously save energy and reduce emissions, which requires the strict supervision of local governments. The impact of external regional environmental protection investment on the local environmental quality may lead to the reduction of environmental protection investment for the effect of regional governments’ ‘competition to the bottom’ and ‘free-riding’ behaviour, which is not conducive to the environmental governance of the region. The greater the spillover effect, the greater the negative effect. Therefore, local governments must choose the strategy of strict supervision to reduce the negative effect.
  (ii) As shown in Figure 2, when $h_{1}=1.3$, it meets the condition that $h_{1}-1>0$; as the positive externality coefficient u and the negative externality coefficient w rise from 0 to 1, the probability of the local government choosing strict supervision strategies decreases rapidly. However, the rate of decline under the influence of the positive externality coefficient gradually falls, and the decline rate under the influence of the negative externality coefficients continues to accelerate. It can be interpreted that high environmental governance benefits mean that environmental policies can effectively motivate local enterprises to consciously save energy and reduce emissions, which has low dependence on the local government’s strict supervision. Clean technology and environmental pollution in external areas may encourage local enterprises to increase investment in energy conservation and emission reduction due to the ‘demonstration effect’ and ‘warning effect’. The larger the spillover effect is, the more positive the effect is, reducing the willingness of local governments to strictly supervise.
(2) Figure 3 illustrates the impact of punishment F and reward B on the choice of the enterprise’s green technology innovation strategy under the condition of other parameters being unchanged. Figure 3 indicates that with the increase in reward and punishment, the probability of an enterprise choosing complete green technology innovation strategies gradually increases, and both of the rising speeds gradually decrease. In practice, reward increases enterprises’ profits, while punishment increases enterprises’ costs. The choice of complete green technology innovation is the best choice to increase revenue and reduce costs for enterprises.
(3) Figure 4 shows the impact of punishment F and reward B on the choice of the local government in choosing strict supervision strategies under the condition of other parameters remaining unchanged. Figure 4 indicates that with the increase in reward, the probability of the local government choosing strict supervision strategies gradually decreases, and both of the rising speeds gradually decline. It can be explained that reward increases the environmental governance costs of local governments and reduces the willingness of local governments to supervise strictly.

The impact of punishment F on the probability of the local government choosing strict supervision strategies will be discussed in two situations:

(i) When $k(1-l)C_{1}-B+(\lambda_{1}-1)e<0$, the profit of local governments is less than 0 when enterprises choose complete green technology innovation and local governments choose strict supervision. With the increase in punishment, the probability of the local government choosing strict supervision strategies gradually increases, and the rising speed gradually decreases. It can be interpreted that the low profit of local governments is led by the great difficulties in regional environmental governance due to the low willingness of enterprises to engage in green technology innovation. Regional environmental governance is highly dependent on governments’ supervision, and a strict supervision strategy can guarantee the incentive effect of punishment mechanisms on enterprises’ green technology innovation behaviour.
(ii) When $k(1-l)C_{1}-B+(\lambda_{1}-1)e>0$, the profit of local governments is greater than 0 when enterprises choose complete green technology innovation and local governments choose strict supervision. With the increase in punishment, the probability of the local government choosing strict supervision strategies gradually falls, and the rate of decline gradually decreases. It can be interpreted that the high profit of local governments is led by fewer difficulties in regional environmental governance due to the high willingness of enterprises to engage in green technology innovation. The effective implementation of punishment mechanisms no longer needs the strict supervision of local governments.

(4) Figure 5 shows the impact of an enterprise’s emission reduction capacity on the choice of its green technology innovation strategy and the local government’s environmental regulation strategy under the condition of other parameters being unchanged. Figure 5 illustrates that as the emission reduction rate rises from 0 to 1, the probability of an enterprise choosing a complete green technology innovation strategy will increase, while the probability of the local government choosing strict supervision strategies will decrease rapidly. This finding has the profound practical meaning that the stronger the enterprises’ emission reduction capability, the greater the willingness of enterprises to save energy and reduce emissions, leading to a decline in the willingness of local governments to strictly supervise.

## 5. Conclusions and Policy Recommendations

### 5.1. Conclusions

From the perspective of local government competition, the spillover of local governments’ investment behaviour has been incorporated into the objective function of the local governments to construct an evolutionary game model between local governments and the enterprises. The following conclusions are obtained.

(1) Under the constraints of environmental regulation, spillover effects have a significant impact on the payment function of local governments, leading to distortions in the implementation of environmental regulations. When the payment function changes, there are four evolutionary stability strategies in the evolutionary game system of local governments and enterprises:
  (I) strict supervision and complete green technology innovation;
  (II) non-strict supervision and complete green technology innovation;
  (III) strict supervision and incomplete green technology innovation; and
  (IV) non-strict supervision and incomplete green technology innovation.
(2) When the environmental benefit of the local governments’ environmental protection investment behaviour is less than the threshold, the spillover of local government investment behaviour may lead to the reduction of regional environmental protection investment for the effect of ‘competition to the bottom’ and ‘free riding behaviour’, which enhances the dependence of the improvement of environmental quality on the strict supervision of local governments. Hence, the probability of local governments choosing the strict supervision strategy increases with the increase in the spillover effect. When the environmental benefit of the local governments’ environmental protection investment behaviour is greater than the threshold, clean technology and environmental pollution in external areas may encourage local enterprises to increase investment in energy conservation and emission reduction for the ‘demonstration effect’ and the ‘warning effect’. This leads to the low dependence of the improvement of environmental quality on the strict supervision of local governments. Hence, the probability of local governments choosing a strict supervision strategy decreases with the increase in the spillover effect of strict supervision.
(3) The reward for enterprises’ complete green technology innovation behaviour increases enterprises’ profits, while punishment for enterprises’ incomplete green technology innovation behaviour increases enterprises’ costs. The choice of complete green technology innovation strategy is the best choice to increase revenue and reduce costs for enterprises. Hence, with the increase in reward and punishment, the probability of enterprises choosing complete green technology innovation strategy gradually increases.
(4) The reward for enterprises’ complete green technology innovation behaviour increases the environmental governance costs of local governments and reduces the willingness of local governments to strictly supervise. The impact of punishment on the probability of the local government choosing strict supervision strategy is uncertain.
(5) The stronger enterprises’ emission reduction capability is, the greater the willingness of enterprises to save energy and reduce emissions, leading to a decline in the willingness of local governments to strictly supervise.

### 5.2. Practical Implications and Limitations

First, the central government should formulate an environmental governance supervision system according to the differences in the environmental awareness and environmental responsibility of residents in different regions, making full use of the ‘demonstration effect’ of regional environmental governance behaviour spillover to produce a positive impact on regional environmental governance decision-making. Within the region, the central government should unify regional environmental management laws, standards, and policy systems, establish regional pollution compensation mechanisms, benefit coordination mechanisms, and ‘green GDP’ competition mechanisms, and improve environmental information sharing mechanisms, joint early warning mechanisms, and demonstration effect mechanisms.

Second, local governments should formulate differentiated reward and punishment mechanisms for green technology innovation in combination with the differences in environmental quality in different regions. Local governments should guide enterprises to actively carry out green technology innovation activities through appropriate fiscal and tax preferential policies and increase support for energy conservation, emission reduction and pollution prevention technology research and development. To form an effective incentive and restraint mechanism for green technological innovation, reward should be increased in areas with better environmental quality and punishment should be strengthened in areas with poor environmental quality.

Third, local governments should formulate differentiated supervision systems based on individual differences in the emission reduction capabilities of different companies. Through incentive means, enterprises with strong emission reduction capacity should be guided to give full play to the ‘demonstration effect’, strengthen the cooperation of regional enterprises in the research and development and utilization of green technology, speed up the elimination of ineffective production capacity, and improve environmental production performance. At the same time, local governments should strengthen the supervision of the energy-saving and emission-reduction behaviours of enterprises with poor emission reduction capabilities, not only to avoid the effect of ‘competition on the bottom line’ among enterprises but also to prevent the transfer of heavily polluting enterprises between different regions.

Based on evolutionary game theory, this paper studies the impact of external government investment spillover on the strategic choice of local governments and enterprises in regional competition and suggests a direction for improving the transmission mechanism of environmental regulation. However, in the construction of the game relationship between environmental regulation subjects, only the key factors in the behavioural characteristics of local governments and enterprises are considered. Future research will combine risk preference, environmental awareness, and social responsibility factors to examine the impact of local government competition on the strategic choice of game players.

## Figures and Tables

**Table 1:** Payment matrix of the game between the local government and the enterprise.

	Strict Supervision	Non-Strict Supervision
Complete green technology innovation	$-k(1-l)+B+h_{2}P-c_{2}$, $k(1-l)-B+(h_{1}-1)P-c_{1}$	$r_{1}B+r_{1}h_{2}P-k(1-l)r_{1}-c_{2}$, $k(1-l)r_{1}-r_{1}B+r_{1}(h_{1}-1)P-r_{1}c_{1}$
Incomplete green technology innovation	$h_{2}P-k(1-r_{2}l)-F-r_{2}c_{2}$, $k(1-r_{2}l)-c_{1}+(h_{1}-1)P+F$	$r_{1}h_{2}P-r_{2}c_{2}-k(1-r_{2}l)r_{1}-r_{1}F$, $k(1-r_{2}l)r_{1}-r_{1}c_{1}+r_{1}(h_{1}-1)P+r_{1}F$

**Table 2:** Expression of the determinant and trace related to the four equilibrium points in system (11).

Equilibriums	tr(A)	det(A)
(0, 0)	$-A_{2}-A_{3}$	$A_{2}A_{3}$
(0, 1)	$A_{1}+A_{2}$	$A_{1}A_{2}$
(1, 0)	$A_{3}+A_{4}$	$A_{3}A_{4}$
(1, 1)	$-A_{1}-A_{4}$	$A_{1}A_{4}$
(x*, y*)	0	$\frac{\left[(1-r_{2})(kl-c_{2})+B+F][kl-k+B+c_{1}+(1-\lambda_{1})e\right]}{(1-r_{1}){\left[kl(1-r_{2})+B+F\right]}^{4}}$

**Table 3:** Local stability of the equilibrium point (Situations 1, 2 and 3).

Equilibrium	Situation 1			Situation 2			Situation 3		
	trJ	detJ	Stability	trJ	detJ	Stability	trJ	detJ	Stability
(0, 0)	Uncertain	−	Saddle point	−	+	ESS	Uncertain	−	Saddle point
(0, 1)	+	+	Instability	+	+	Instability	Uncertain	−	Saddle point
(1, 0)	−	+	ESS	Uncertain	−	Saddle point	+	+	Uncertain
(1, 1)	Uncertain	−	Saddle point	Uncertain	−	Saddle point	−	+	ESS

Note: ESS refers to Evolutionary Stability Strategy, det(A) and tr(A) respectively refers to the expression of determinant and trace in the evolutionary game system.

**Table 4:** Local stability of the equilibrium point (Situations 4, 5 and 6).

Equilibrium	Situation 4			Situation 5			Situation 6		
	trJ	detJ	Stability	trJ	detJ	Stability	trJ	detJ	Stability
(0, 0)	Uncertain	−	Saddle point	+	+	Instability	+	+	Instability
(0, 1)	Uncertain	−	Saddle point	Uncertain	−	Saddle point	Uncertain	−	Saddle point
(1, 0)	Uncertain	−	Saddle point	Uncertain	−	Saddle point	−	+	ESS
(1, 1)	Uncertain	−	Saddle point	−	+	ESS	Uncertain	−	Saddle point

**Table 5:** Local stability of the equilibrium point (Situations 7, 8 and 9).

Equilibrium	Situation 7			Situation 8			Situation 9		
	trJ	detJ	Stability	trJ	detJ	Stability	trJ	detJ	Stability
(0, 0)	−	+	ESS	Uncertain	−	Saddle point	Uncertain	−	Saddle point
(0, 1)	Uncertain	−	Saddle point	−	+	ESS	−	+	ESS
(1, 0)	Uncertain	−	Saddle point	+	+	Instability	Uncertain	−	Saddle point
(1, 1)	+	+	Instability	Uncertain	−	Saddle point	+	+	Instability

## Data Availability

Data is contained within the article.

## Appendix A

**Proposition** **A1.**
*When the environmental protection investment performance of local governments is relatively low ($h_{1}-1<0$), enhancing the positive and negative externality coefficients of external government investment behaviour is conducive to increasing the probability that the local government will choose strict supervision. When the environmental protection investment performance of local governments is relatively large ($h_{1}-1>0$), reducing the positive and negative externality coefficients of external government investment behaviour is conducive to increasing the probability that the local government will choose strict supervision. In addition, compared to the positive externality coefficient, the negative externality coefficient has a greater impact on the probability of the local government choosing strict supervision.*

**Proposition** **A2.**
*Enhancing the reward for enterprises’ complete green technology innovation is conducive to increasing the probability of the enterprises choosing the complete green technology innovation strategy, while it suppresses the probability of the local government choosing the strict supervision strategy. Enhancing punishment for enterprises’ incomplete green technology innovation behaviour is conducive to increasing the probability of enterprises choosing the complete green technology innovation strategy, but its impact on the probability of the local government choosing the strict supervision strategy is uncertain due to the limitations of many factors.*

**Proposition** **A3.**
*The stronger the enterprise’s emission reduction capacity, the stronger its environmental awareness and responsibility and the greater the investment in emission reduction technology research and development.*

**Proof of Proposition** **A1.** Please see the Appendix A. Figure 1d shows that the larger the sum of areas I and III, the greater the probability that the local government will choose a strict regulatory strategy, and the larger the sum of areas I and II, the greater the probability that enterprises will choose a complete green technological innovation strategy. The sum of areas I and III can be expressed as $S_{\left(I+III\right)}=x*=\frac{k(1-r_{2}l)+F-C_{1}+(h_{1}-1)P}{kl(1-r_{2})+B+F}$. The derivative of the $S_{\left(I+III\right)}$ equation of u and w can be calculated as follows. ∂S(I+III)∂u=(h1−1)∂P∂ukl(1−r2)+B+F, ∂S(I+III)∂w=(h1−1)∂P∂wkl(1−r2)+B+F. The derivative of the e equation of u and w can be calculated as follows. ∂P∂u=−αβm(1−w)(β+βu+α−αw)2<0, ∂P∂w=−αβm(1+u)(β+βu+α−αw)2<0. It is always valid that $kl(1-r_{2})+B+F>0$, so when $h_{1}-1<0$, 0<∂S(I+III)∂u<∂S(I+III)∂w. When, $h_{1}-1>0$, 0>∂S(I+III)∂u>∂S(I+III)∂w. Proposition A1 has been proven. □

**Proof of Proposition** **A2.** The sum of areas I and II can be expressed as $S_{\left(I+II\right)}=1-y*=\frac{1}{1-r_{1}}-\frac{c_{2}(1-r_{2})}{\left(1-r_{1})[kl(1-r_{2})+B+F\right]}$. The derivative of the $S_{\left(I+II\right)}$ equation of B and F can be calculated as follows. ∂S(I+II)∂B=∂S(I+II)∂F=c2(1−r2)(1−r1)[kl(1−r2)+B+F]2. The derivative of the $S_{\left(I+III\right)}$ equation of B and F can be calculated as follows. ∂S(I+III)∂B=(1−h1)P−F+C1−k(1−r2l)[kl(1−r2)+B+F]2, ∂S(I+III)∂F=C1+B−(h1−1)P−k(1−l)[kl(1−r2)+B+F]2. It is always valid that ∂S(I+II)∂B=∂S(I+II)∂F>0. We know that $k(1-r_{2}l)+F-C_{1}-(1-h_{1})P>0$ for that $S_{\left(I+III\right)}>0$; hence, it is always valid that ∂S(I+III)∂B<0. From the derivative of the $S_{\left(I+III\right)}$ equation of F, we know that only when $C_{1}+B-(h_{1}-1)P-k(1-l)>0$ can ∂S(I+III)∂F>0; otherwise, ∂S(I+III)∂F<0. Proposition A2 has been proven. □

**Proof of Proposition** **A3.** The derivative of the $S_{\left(I+II\right)}$ equation of l can be calculated as follows: ∂S(I+II)∂l=kc2(1−r2)2(1−r1)[kl(1−r2)+B+F]2. The derivative of the $S_{\left(I+III\right)}$ equation of l can be calculated as follows: ∂S(I+III)∂l=k{(1−r2)[C1−(h1−1)P−k]−r2B−F}[kl(1−r2)+B+F]2. It is always valid that ∂S(I+II)∂l>0. We can see that $C_{1}-(h_{1}-1)P<k(1-r_{2}l)+F$ for that $S_{\left(I+III\right)}>0$, for which we can obtain $(1-r_{2})[C_{1}+(h_{1}-1)P-k]<r_{2}B+F$. Hence, ∂S(I+III)∂l<0. Proposition A3 has been proven. □
